# Loop-Mediated Amplification Accelerated by Stem Primers

**DOI:** 10.3390/ijms12129108

**Published:** 2011-12-08

**Authors:** Olga Gandelman, Rebecca Jackson, Guy Kiddle, Laurence Tisi

**Affiliations:** Lumora Ltd., Unit 4, Cambridgeshire Business Park, Bartholomews Walk, Ely, Cambridgeshire CB7 4EA, UK; E-Mails: r.harrall@lumora.co.uk (R.J.); g.kiddle@lumora.co.uk (G.K.); l.tisi@lumora.co.uk (L.T.)

**Keywords:** stem primers, isothermal amplification, loop-mediated amplification, *in vitro* diagnostics, *Clostridium difficile*, *Listeria monocytogenes*, HIV

## Abstract

Isothermal nucleic acid amplifications (iNAATs) have become an important alternative to PCR for *in vitro* molecular diagnostics in all fields. Amongst iNAATs Loop-mediated amplification (LAMP) has gained much attention over the last decade because of the simplicity of hardware requirements. LAMP demonstrates performance equivalent to that of PCR, but its application has been limited by the challenging primer design. The design of six primers in LAMP requires a selection of eight priming sites with significant restrictions imposed on their respective positioning and orientation. In order to relieve primer design constraints we propose an alternative approach which uses Stem primers instead of Loop primers and demonstrate the application of STEM-LAMP in assaying for *Clostridium difficile*, *Listeria monocytogenes* and HIV. Stem primers used in LAMP in combination with loop-generating and displacement primers gave significant benefits in speed and sensitivity, similar to those offered by Loop primers, while offering additional options of forward and reverse orientations, multiplexing, use in conjunction with Loop primers or even omission of one or two displacement primers, where necessary. Stem primers represent a valuable alternative to Loop primers and an additional tool for IVD assay development by offering more choices for primer design at the same time increasing assay speed, sensitivity, and reproducibility.

## 1. Introduction

Loop-mediated amplification (LAMP) is an emerging alternative to PCR for *in vitro* diagnostic (IVD) applications in clinical, industrial and veterinary applications. LAMP performance compares to that of PCR in terms of sensitivity and specificity [1–4]. Unlike PCR, LAMP does not require thermocycling and is not reliant on confirmatory probes for high specificity. Real-time detection is possible through fluorescence, turbidity or bioluminescence; the latter allows uniquely simple and robust hardware to be employed [1,5,6]. This facet is especially important in bringing the many benefits of molecular diagnostics to market segments where the complexity and cost of reagents and instrumentation for methods such as PCR have prevented more extensive applications of molecular diagnostics.

**Figure 1:**
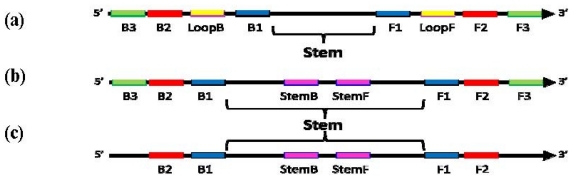
Schematic diagram showing locations of priming sites and the “Stem” in loop-mediated amplification (LAMP) with Loop primers (**a**), LAMP with Stem primers (**b**) and LAMP with Stem primers but without “Displacement primers” (**c**).

LAMP was originally invented and formulated as an isothermal amplification with the strict requirement for four primers: two loop-generating primers (FIP and BIP comprising F1, F2 and B1, B2 priming sites, correspondingly) and two “Displacement primers” (F3 and B3) [7]. However, in this manifestation the LAMP technology was far too slow for the majority of practical applications. In order to increase the speed of LAMP-based assays the inventors of LAMP came up with additional “Loop primers” which, when added in conjunction with the other primers used in LAMP, resulted in significantly faster assays [8]. Currently, the commonly used manifestation of LAMP requires a total of six primers (Figure 1a): two loop-generating primers, two displacement primers and two “Loop primers” (LoopB and LoopF). Primer design for LAMP assays thus requires the selection of eight separate regions of a target nucleic acid sequence (the FIP and BIP primers encompass two primer binding sites each), with the BIP/FIP and Loop primers having significant restrictions on their positioning respective to each other. “Loop primers” must be positioned strictly between the B2 and B1 sites and the F2 and F1 sites, respectively, and must be orientated in one particular direction. Further, significant care must be taken in primer design to avoid primer-dimers between the six primers needed (especially difficult as the FIP and BIP primers are generally greater than 40 nucleotides long). As a consequence, LAMP primer design is extremely challenging, especially when targeting highly polymorphic markers and sequences containing complex secondary structure.

The investigators report here an alternative to “Loop primers”, an additional tool *per se*, which offers a broader range of primer design options since they can be positioned with fewer restrictions than apply to “Loop primers”. These primers, known as “Stem primers” (as they target the “Stem” in LAMP amplicon) when used in addition to loop-generating and displacement primers offer similar benefits in speed and sensitivity to the Loop primers. This beneficial effect of Stem primers is surprising as they do not bind to the single-stranded DNA loops, which define the very nature of the LAMP technology [7,9]. Stem primers can be employed in either orientation, do not require either the B2/B1 or F2/F1 sites to be a specific distance apart, can be multiplexed, and allow the F1 and B1 sites to be positioned further from each other than in LAMP (Figure 1b).

## 2. Results and Discussion

All experimental results presented in the manuscript were obtained using Lumora’s bioluminescence-based proprietary BART technology for monitoring amplification in real-time [5]. BART allows qualitative and quantitative detection of amplification. BART in combination with LAMP has demonstrated a performance equivalent to that of real-time PCR. In BART the presence of a nucleic acid target is characterized by a bright flash of light. Time-to-peak of the flash is inversely proportional to the amount of target present in the assay (a relationship similar to that observed between the Ct value and the copy number in PCR).

### 2.1. Stem Primers as an Alternative to Loop Primers for Accelerating Amplification of DNA and RNA in LAMP

#### 2.1.1. Genomic DNA Target Amplification

**Figure 2:**
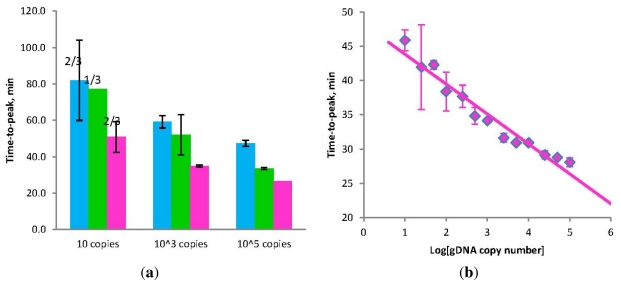
(**a**) Amplification times for LAMP without Loop primers (blue) and STEM-LAMP with Stem primers instead of Loop primers (pink) for 10, 1000 and 100,000 copies of genomic *Clostridium difficile* DNA target; STEM-LAMP without displacement primers for the same number of copies is shown in green; (**b**) a six log dilution series of *Clostridium difficile* toxin B gene using STEM-LAMP (all dilutions were measured and detected in triplicate unless indicated otherwise). Final primers concentrations were: Cd-BIP, Cd-FIP, Cd-StemB and Cd-StemF—1.6 μM each, Cd-DisplB and Cd-DisplF—0.4 μM each. Each set of data included three no template controls (NTCs), that remained clean throughout the duration of the assay and were not associated with any time-to-peak value (hence not shown graphically). Error bars represent standard deviations of triplicate measurements.

The ability of Stem primers to efficiently accelerate LAMP assays was exemplified on the toxin B gene of *Clostridium difficile.* A comparison was made between LAMP in the absence of Loop primers and LAMP with Stem primers instead of Loop primers, referred to herein as STEM-LAMP (Figure 2a). LAMP, without Loop primers, detected *Clostridium difficile* genomic DNA down to 10 copies but taking approximately 80 minutes to do so. Addition of Stem primers accelerated the amplification significantly, detecting 10 copies of *Clostridium difficile* genomic DNA about 30 minutes faster than in their absence. Hence, Stem primers can significantly accelerate LAMP assays.

Remarkably, the use of Stem primers allowed effective LAMP assays to be performed *in the absence of displacement primers* (green bars in Figure 2a). This is surprising as displacement primers were postulated to be critical to the mechanism of the LAMP reaction [9], yet, with Stem primers, efficient amplification occurs in the absence of displacement primers.

A dilution series of *Clostridium difficile* genomic DNA was assayed using the STEM-LAMP in the presence of 500 ng carrier DNA per 20-μL test to assess dynamic range and linearity (Figure 2b). A strong linear correlation was observed in semi-logarithmic coordinates (Time-to-peak versus Copy number) within the dilution range of 10 and 100,000 copies (Figure 2b). STEM-LAMP demonstrated fast and sensitive performance equivalent to that of the FDA approved LAMP assay from Meridian (16 CFU/test in 60 min) [10]. Hence, STEM-LAMP is not only fast and suitable for qualitative applications but can be also used in quantitative assays.

#### 2.1.2. Plasmid DNA Target Amplification

To check that the effect of Stem primers was generic, and not a quirk of the *Clostridium difficile* target described above, several other markers were analyzed.

**Figure 3:**
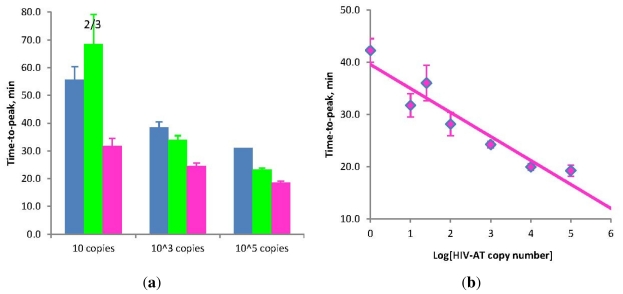
(**a**) Amplification times for LAMP without Loop primers (blue) and STEM-LAMP with Stem primers instead of Loop primers (pink) for 10, 1000 and 100,000 copies of the HIV-AT construct (restricted with Kpn1 and pre-denatured); STEM-LAMP without displacement primers for the same copy number is shown in green; (**b**) A six log dilution series of the HIV-AT construct using STEM-LAMP (all dilutions were measured and detected in triplicate unless indicated otherwise). Final primers concentrations were: sHIV-BIP, sHIV-FIP, HIV-StemB and HIV-StemF—1.6 μM each, HIV-DisplB and HIV-DisplF—0.4 μM each. Each set of data included three NTCs that remained clean throughout the duration of the assay and were not associated with any time-to-peak value (hence not shown graphically). Error bars represent standard deviations of triplicate measurements.

In one case a plasmid containing a cloned fragment of the HIV polymerase gene (HIV-AT) was assayed in the range of 10 and 100,000 copies per test. As in the previous example, using Stem primers led to a significant acceleration of amplification, which was particularly noticeable at the lowest copy number (Figure 3a). Again, remarkably, the presence of Stem primers allowed effective amplification to occur even with the omission of displacement primers.

To evaluate the quantitative potential of STEM-LAMP a dilution series of the plasmid in the range of 1 to 100,000 copies per 20-μL test was amplified in the presence of 500 ng carrier DNA. Linear correlation between the average time-to-peak and the copy number was observed in semi-logarithmic coordinates across the whole range (Figure 3b). STEM-LAMP can be used for qualitative and quantitative testing of plasmid targets.

#### 2.1.3. *In vitro* Transcribed RNA (IVT RNA) Target in Reverse Transcription LAMP (RT-LAMP)

**Figure 4:**
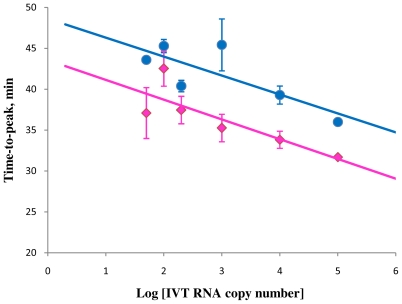
Correlation between amplification times in RT-STEM-LAMP (pink) and RT-LAMP (blue) and the copy number of IVT RNA in semi-logarithmic coordinates (all dilutions were measured and detected in triplicate unless indicated otherwise). Final primers concentrations were: sHIV-BIP, sHIV-FIP, HIV-StemB, HIV-StemF, HIV-BIP, HIV-FIP, HIV-LoopB and HIV-LoopF—1.6 μM each, HIV-DisplB and HIV-DisplF—0.4 μM each. Each set of data included three NTCs that remained clean throughout the duration of the assay and were not associated with any time-to-peak value (hence not shown graphically). Error bars represent standard deviations of triplicate measurements.

To assess the performance of STEM-LAMP on RNA targets, a comparison was made between RT-LAMP (in the presence of Loop primers) and RT-STEM-LAMP using a range of HIV IVT RNA copy number in a one-tube assay. Both systems performed much better in the presence of either Loop or Stem primers than in their absence (data nor shown) and detected down to 50 copies RNA within 60 min (Figure 4). Both demonstrated linear correlation between amplification time and (the logarithmic of) copy number with similar slopes. The speed of the RT-STEM-LAMP was comparable to the RT-LAMP assay, in fact, slightly faster.

Stem primers can be used for acceleration of RNA target amplification as well as for DNA targets. The increase in speed with Stem primers is fully comparable to that achieved with Loop primers. The linearity of the response is typical for both systems and is a useful feature for quantitative assays.

### 2.2. Stem Primers Used in Conjunction with Loop Primers for Accelerating Amplification of DNA in LAMP

To assess whether Loop and Stem primers could work in an additive fashion to increase the speed of amplification, a model plasmid system was designed based on the *Listeria monocytogenes* inlA gene (inlA-AT).

**Figure 5:**
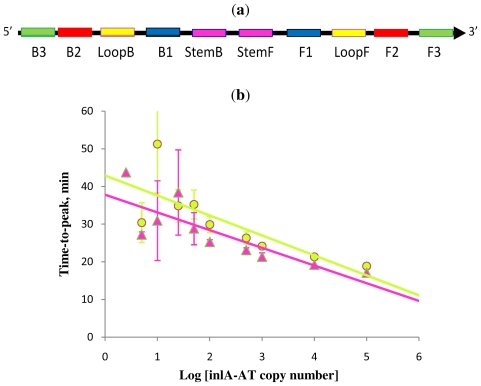
Schematic diagram of all priming sites used in Stem-accelerated LAMP using Loop primers (LAMP-LOOP) (a); comparison of LAMP-LOOP with inlA-AT target in the absence (lime) and presence (pink) of Stem primers (b) over a six log dilution series (all dilutions were measured and detected in triplicate unless indicated otherwise). Final primers concentrations were: Lm-BIP, Lm-FIP, Lm-StemB, Lm-StemF, Lm-LoopB, Lm-LoopF, Lm-DisplB and Lm-DispIF—0.8 μM each. Each set of data included three NTCs that remained clean throughout the duration of the assay and were not associated with any time-to-peak value (hence not shown graphically). Error bars represent standard deviations of triplicate measurements.

A schematic representation of the target region containing all ten priming sites is shown in (Figure 5a). A comparison of speed and sensitivity for LAMP, using Loop primers, and LAMP using both Loop and Stem primers was carried out using a cloned target sequence in the range of 1 to 100,000 copies (Figure 5b). LAMP using Loop primers was shown to be very fast and sensitive with the limit of detection equal to 5 copies per 20-μL test in 60 min and the limit of quantification equal to 50 copies in 40 min. In the presence of Stem primers the test demonstrated significantly improved performance allowing quantification down to 50 copies within 30 min.

Thus, where sequences allow, Stem primers can be used in conjunction with Loop primers to obtain assays with improved speeds and hence shorter time-to-result. This may be particularly useful in applications where time-to-result is a crucial factor, e.g., in near-patient testing.

### 2.3. Beneficial Features of Stem Primers

#### 2.3.1. Multiplexing Stem Primers for Further Increase of Amplification Speed

Whilst Stem primers can be used in conjunction with Loop primers to give an additive positive effect, it may, in practice, be difficult to find 10 primer binding sites on a target of interest and to accommodate the strict interdependencies of the Loop and FIP/BIP primers in particular. An alternative approach is to omit Loop primers and introduce binding sites for additional Stem primers instead. The latter have fewer restrictions on their positioning relative to the FIP/BIP primers.

**Figure 6:**
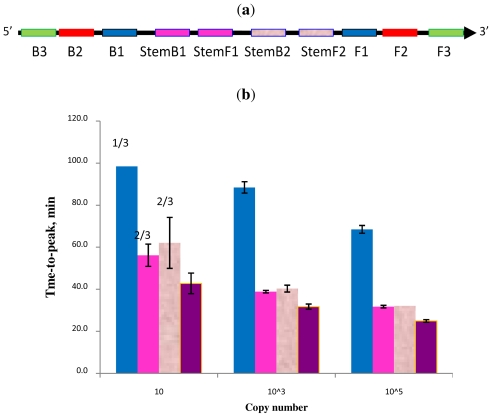
Schematic diagram of all priming sites used in STEM-LAMP with two sets of multiplexed Stem primers (**a**): a comparison of LAMP performance in the absence and presence of Stem primers for 10, 1000 and 100,000 copies of genomic *Clostridium difficile* DNA target (**b**): in the absence of Stem primers—blue, in the presence of Stem primers set 1—pink, Stem primers set 2—rose, multiplexed Stem primers sets 1 and 2—purple (all dilutions were measured and detected in triplicate unless indicated otherwise). Final primers concentrations were: Cd-BIP, mCd-FIP, Cd-StemB1, Cd-StemF1, Cd-StemB2 and Cd-StemF2—1.6 μM each, Cd-DisplB and mCd-DisplF—0.4 μM each. Each set of data included three NTCs that remained clean throughout the duration of the assay and were not associated with any time-to-peak value (hence not shown graphically). Error bars represent standard deviations of triplicate measurements.

Two sets of Stem primers were designed for the *Clostridium difficile* toxin B gene; a schematic representation of the priming sites is shown in (Figure 6a). Amplification was performed in the absence of Stem primers and with either one or both sets of Stem primers at 10, 1000 and 100,000 copies of genomic DNA per test (Figure 6b).

In agreement with the results presented above LAMP without any Stem primers was the slowest and detected only one out of three replicates for 10 copies of DNA within 100 min (Figure 2a). In the presence of either Stem primers set 1 or 2 amplification was much faster and two out of three 10 copies were detected in less than 70 min. In the presence of both sets of Stem primers 10 copies of the target were detected in triplicate, in less than 50 min and with better reproducibility. The initial experiments showed that multiplexing of Stem primers had no adverse effect on amplification while gave an additive benefit with further increases in amplification speed and improved reproducibility, particularly at low copy number. Similar multiplexing of Loop primers in LAMP has never been reported and may be practically impossible because of the limitations imposed upon the length of B2-B1 and F2-F1 regions.

In this case the “Stem” region on the target DNA sequence may be much longer than that usually selected for LAMP assay designs, yet rapid detection is still achieved. This suggests that Stem primers further facilitate primer design by allowing the B1 and F1 sites to be situated further apart.

#### 2.3.2. Flexibility with Orientation of Stem Primers

In all presented examples Stem primers were used in equivalent orientations to Loop primers. To see how sensitive Stem and Loop primers were to orientation, a comparison was made between Stem and Loop primers with primers designed in both forward and reverse orientations.

**Figure 7:**
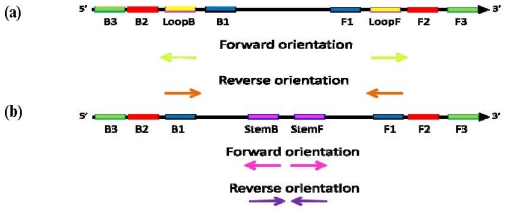

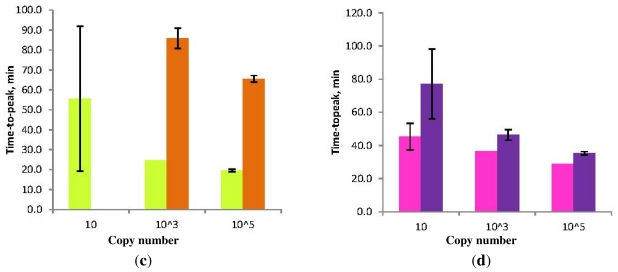
Schematic diagram of the “forward” (conventional) and “reverse” orientation of Loop primers in LAMP-LOOP (**a**); Stem primers in STEM-LAMP (**b**) and comparison of LAMP-LOOP with Loop primers in conventional (lime) and reverse (orange) orientation (**c**) and STEM-LAMP with Stem primers in conventional (pink) and reverse (purple) orientation (**d**) for 10, 1000 and 100,000 copies of inlA-AT construct (all dilutions were measured and detected in triplicate unless indicated otherwise). Final primers concentrations were: Lm-BIP, Lm-FIP, Lm-StemB, Lm-StemF, Lm-LoopB, Lm-LoopF, rLm-StemB, rLm-StemF, rLm-LoopB, rLm-LoopF, Lm-DisplB and Lm-DisplF—0.8 μM each. Each set of data included three NTCs that remained clean throughout the duration of the assay and were not associated with any time-to-peak value (hence not shown graphically). Error bars represent standard deviations of triplicate measurements.

A comparison of LAMP-LOOP and STEM-LAMP was carried out in combination with Loop and Stem primers, respectively, in conventional and reverse orientation (Figure 7). The inlA-AT construct was used as a model system at 10, 1000 and 100,000 copies. The replacement of Stem primers in orientation identical to that conventionally used for Loop primers by Stem primers with the reverse orientation caused a modest slow down in amplification but 10 copies were still detected in triplicate in less than 80 min (Figure 7a). When a similar comparison was implemented for Loop primers, Loop primers in reverse orientation had a detrimental effect on the speed of LAMP amplification, which resulted in 1000 copies peaking at around 80 min 10 copies were not detected when the assay ended after 100 min (Figure 7b).

**Figure 8:**
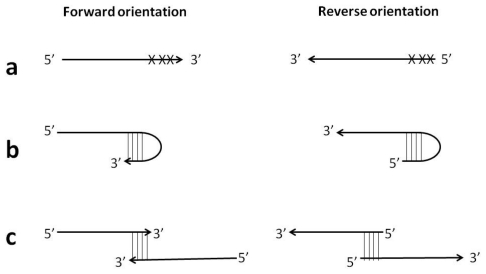
Examples of the reverse primer orientation feasibility: (**a**) avoidance of mismatches in close proximity to 3′-end; (**b**) avoidance of hairpin formation at 3′-end; (**c**) avoidance of self- or hetero-primer-dimerization at 3′-end.

In this case, Stem primers proved considerably more robust to their orientation than Loop primers. A possibility to use primers in either orientation is an attractive feature in primer design involving targets with poor sequence complexity, high GC-content, low sequence conservation or complex secondary structure, e.g., prone to hairpin formation or repeats potentially leading to primer-dimer interactions and non-specific amplification (Figure 8).

### 2.4. Mechanism of Action of Stem Primers

The fact that Stem primers can so effectively accelerate loop-mediated amplification in the absence of Loop primers is, at first, surprising as the Stem primers do not target the very “Loops” which the LAMP technology is based on. Presumably, Stem primers are able to prime off the newly generated amplicon as it is made transiently single-stranded during DNA replication. However, the lifetime of such single-stranded amplicon is likely to be very short unlike the more stable “loops” to which the Loop primers and FIP/BIP primers are able to bind.

**Figure 9:**
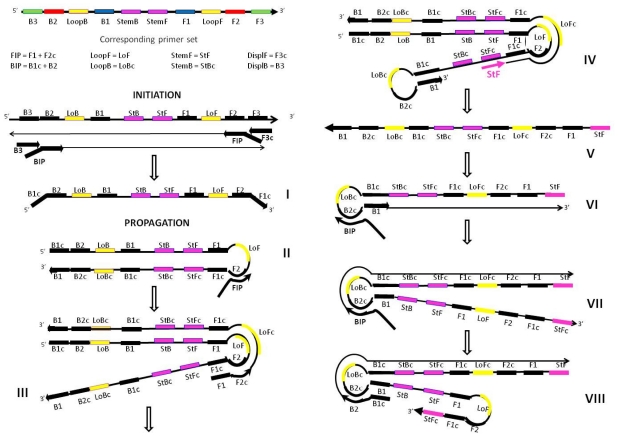
Proposed mechanism for the acceleration of LAMP amplification with Stem primers showing the potential location of all priming sites essential for the propagation of the amplicon (only one strand is shown for example purposes). Structures III, V and VIII depict the distinctive features of Stem primer’s action. Structure III has a transiently single strand available for the productive binding of the StemF primer. Structure V is the amplicon produced as a result of the extension of StemF primer. Structure VIII is self-extending through extra strong intra-molecular self-priming.

However, when one looks in detail at the LAMP mechanism a number of differences between the action of the Stem primers and the Loop primers becomes apparent, which may explain why Stem primers give better than expected performance. A speculative mechanism depicting the action of Stem primers in loop-mediated amplification is presented in Figure 9. Initiation stage that leads to the formation of Structure I capable of generating a loop at 3′-end and self-extending into a pan-handle (Structure II) involves only loop-generating and displacement primers and is therefore identical between the Loop- and Stem-accelerated LAMP. The same is true for the first round of annealing and extension of FIP primer causing the displacement of the opposite strand (Structure III). Once this single strand is released, it provides a binding site for StemF primer; at that time in the amplification process there is still no binding site available for either of the Loop primers. StemF primer anneals to the transiently single strand (Structure III) and extends, displacing FIP-initiated amplicon (Structure IV). Therefore in the process of amplification Stem primers anneal and extend giving rise to a recopiable amplicon earlier than Loop primers.

Structure IV can also extend through intra-molecular self-priming at 3′-end displacing the StemF-initiated amplicon (Structure V). Structure V is unique to Stem-accelerated LAMP: it contains the entire binding sites for the BIP/FIP primers and facilitates immediate propagation of new amplicon chains (Figure 9, structure VI). Structure VI plays a role similar to that of Structure II: it provides a single-stranded loop for the annealing and extension of BIP primer which releases the opposite strand VII. The freed 3′-end in the latter is delimited by the re-copied StemF priming site and allows for extra strong intra-molecular self-priming (Figure 9, structure VIII) which does not occur in loop-accelerated LAMP. The amplification then proceeds into forming concatemers.

In contrast, for Loop primers, when Structure IV is formed, no site is yet available for a Loop primer to anneal and produce a recopiable amplicon. A priming site for productive binding of Loop primer is formed only in the following round of the amplification. Further, when Loop primers anneal to single-stranded loops and extend, the recopied reverse chains do not include the entire binding sites for either BIP/FIP primers at 3′-end of the amplicon or for allowing intra-molecular self-priming.

**Figure 10:**
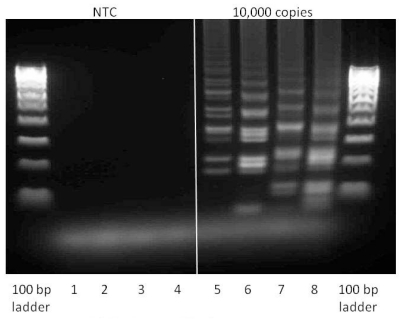
Gel analysis of positive (containing 10^4^ copies inlA-AT) and negative samples (reaction conditions are as described in Section 2.2). 1 μL of each reaction mix was loaded into each well and run for 2 h at 75 V on the 2% agarose gel. Lanes 1 and 5—LAMP with Lm-BIP, Lm-FIP, Lm-DisplB and Lm-DisplF. Lanes 2 and 6—LAMP-LOOP with Lm-BIP, Lm-FIP, Lm-DisplB, Lm-DisplF, Lm-LoopB, Lm-LoopF. Lanes 3 and 7—STEM-LAMP with Lm-BIP, Lm-FIP, Lm-DisplB, Lm-DisplF, Lm-StemB, Lm-StemF. Lanes 4 and 8—LAMP with Stem and Loop primers: Lm-BIP, Lm-FIP, Lm-DisplB, Lm-DisplF, Lm-LoopB, Lm-LoopF, Lm-StemB, Lm-StemF.

The differences in mechanism between Loop- and Stem-accelerated LAMP are reflected in the pattern of bands revealed by gel electrophoresis (Figure 10). Lanes 1–4 containing no-template controls did not exhibit any amplification products proving that no non-specific amplification resulting from primer-dimerization was observed. LAMP in its original manifestation (containing only loop-generating and displacement primers) demonstrated a typical pattern of repeating duplicate bands that reflected concatamerization of the amplicon (lane 5). Addition of Loop primers caused no major change in the size of the duplicated bands but introduced an additional repeating band of the smaller size (lane 6). Addition of Stem primers resulted in the increased size of the main bands, suggesting the production of a longer amplicon delimited by Stem priming sites (lane 7). A combination of all eight primers revealed a pattern resembling a superimposition of those seen in lanes 6 and 7 (lane 8).

### 2.5. Flexibility of Primer Design in STEM-LAMP

Any LAMP assay requires a selection of target and at least eight specific priming sites for the design of six primers. There is a firm set of recommendations and rules to be followed in the target selection and primer design of LAMP systems accelerated by Loop primers, which includes range options, distances between priming sites and orientation of primers [11]. LAMP primer design is a challenging process with limited choices, particularly difficult when highly polymorphic sequences are targeted. Stem primers offer more flexibility and significantly relax those limitations. Firstly, there is no restriction on the proximity of B2-B1 and F1-F2 sites as there is no need to position a Loop primer. Secondly, there is less restriction on the length of the stem, *i.e.*, distance between B1 and F1 sites, as multiple Stem primer sets can be designed to accommodate longer stems, when longer sequences are targeted. Further more detailed investigation of the allowed maximum length of the stem is currently underway. Thirdly, unlike Loop primers that strictly require a particular orientation, Stem primers can be designed and used in either orientation in agreement with the mechanism proposed above. Fourthly, if the target region is short and cannot accommodate all eight binding sites, it is possible to sacrifice one or both priming sites for Displacement primers, *i.e.*, B3 and F3, as STEM-LAMP demonstrated high sensitivity even in their absence. Preliminary results (not shown) indicated that the latter is equally possible in LAMP-LOOP though it contradicts the postulated mechanism for LAMP [7,9].

## 3. Experimental Section

### 3.1. Materials and Reagents

Oligonucleotides (HPLC grade) were synthesized by Eurofins MWG Operon (Ebersberg, Germany). High purity genomic DNA isolated from *Clostridium difficile* strain 630 was obtained from LGC Standards (Teddington, UK). *Listeria monocytogenes* artificial construct (inlA-AT) was made by Top Gene Technologies (Montreal, Canada) and HIV artificial construct (HIV-AT) was made by Eurofins MWG Operon. BARTMaster and RT-BARTMaster are lyophilized presentations of Lumora’s proprietary generic reagents (Lumora Ltd., Ely, UK) containing all ingredients required for BART detection and Bst-based isothermal amplification of DNA or AffinityScript reverse transcriptase-based amplification of RNA respectively [5]. They are resuspended in “Lumopol” buffered diluent and require an addition of primers and target. BARTMaster, RT-BARTMaster and “Lumopol” were produced in-house. Salmon sperm DNA and yeast tRNA were obtained from Invitrogen (Paisley, UK), RiboLock RNase inhibitor from Fermentas (part of Thermo Fisher Scientific, Loughborough, UK), molecular grade water from Sigma-Aldrich (Dorset, UK).

### 3.2. Template Selection and Primer Design

Sequences of oligonucleotides designed for *Clostridium difficile* targeting a toxin B gene sequence (Genbank accession number X92982) are shown below (Table 1).

A 326 base-pair HIV artificial template (HIV-AT) delimited by Sp6-promoter at the 3′-end and ClaI-restriction site at the 5′-end was cloned into the pCR2.1 vector using TOPO-TA kit (Invitrogen, Paisley, UK). The insert reproduces a unique sequence from HIV-1polymerase gene (Genbank accession number K02013.1; positions 3781–4080). RNA was *in vitro* transcribed from the pCR2.1 construct using AmpliScribe™ T7 High Yield Transcription Kit (EPICENTRE Biotechnologies, Madison, WI, USA) according to the manufacturer’s recommendations. To achieve full removal of DNA the mixture was treated twice with RNAse-free DNAse. Quality and concentration of RNA preparations was assessed spectrophotometrically using a Nanodrop spectrophotometer (Thermo Scientific, Wilmington, DE, USA). Sequences of oligonucleotides designed for HIV-AT are shown below (Table 2).

A 273 base-pair artificial template (inlA-AT) was cloned into the pLSvector at the position of HpaI restriction site. The insert reproduces a unique sequence from *Listeria monocytogenes* internalin A gene (Genbank accession number NC_011660; positions 1485–1757). Sequences of oligonucleotides designed for inlA-AT are shown below (Table 3).

### 3.3. BART Assay Formulations and Protocols

All amplifications were monitored using Lumora’s proprietary technology (Lumora Ltd., Ely, UK), a real-time bioluminescent assay in real-time (BART) [5].

#### 3.3.1. LAMP-BART Assay

All DNA amplifications were carried out using lyophilized BARTMaster resuspended in “Lumopol” buffer with different combinations of primers added to the concentrations indicated for each experiment individually [5]. BART assays were run in 20 μL total volume containing 15 μL reagent mix with 5 μL added target solution. Final concentrations of all ingredients in the assay were: 300 μM each dNTP, 0.16 U/μL Bst DNA polymerase large fragment, 100 μg/mL luciferin, 100 μM adenosine- 5′-*O*-phosphosulfate, 0.5 U/mL ATP sulfurylase, 5.6–6.2 μg/mL Ultra*-*Glo™ recombinant luciferase, 60 mM KCl, 0.4 mg/mL polyvinylpyrrolidone, 10 mM DTT, 20 mM tris-HCl (pH 8.8 at room temperature), 2 mM MgSO_4_, 10 mM (NH_4_)_2_SO_4_, 0.1% Triton X-100. Reaction mixtures were covered with mineral oil to prevent evaporation. Where indicated target was pre-denatured for 5 min at 95 °C and its dilutions were made in molecular grade water containing 100 ng/μL salmon sperm DNA. Assays were run in triplicate at 60 °C for 100 min.

#### 3.3.2. RT-LAMP-BART Assay

All RNA amplifications were carried out using lyophilized RT-BARTMaster resuspended in “Lumopol” buffer with 1.6 μM each Lamp, Stem and Loop primer and 0.4 μM each displacement primer. Protocol and final concentrations in the assay were identical to the described above for LAMP-BART but with 0.05 μL/assay AffinityScript reverse transcriptase included in the lyophilized reagent. Reaction mixtures were covered with mineral oil to prevent evaporation. *In vitro* transcribed RNA (IVT RNA) was pre-denatured for 10 min at 70 °C and its dilutions were made in molecular grade water containing 20 ng/μL yeast tRNA and 0.2 U/μL RiboLock RNase inhibitor. Assays were run in triplicate at 47 °C for 20 min followed by 60 °C for 80 min.

#### 3.3.3. Hardware for Measuring BART Assays

All LAMP-BART assays were carried out on a bespoke instrument comprising a PC-controlled 96-well heating block placed beneath a CCD camera in a light-proof enclosure (Syngene, Cambridge, UK) [5].

## 4. Conclusions

Stem primers significantly accelerate LAMP comprised of loop-generating and displacement primers only. They can be used on their own or synergistically with other Stem primers or even Loop primers. Addition of Stem primers into LAMP has a positive effect on both speed and sensitivity. In some cases they improve reproducibility at low copy number.

The action of Stem primers can be rationalized via the proposed mechanism of LAMP. They anneal to transiently single-stranded regions of the amplicon and recopy the entire binding sites for the BIP/FIP primers. An additional unique feature is the extra strong intra-molecular self-priming when Stem primers delimit amplicon.

Positioning of Stem primers is less constrained than that of Loop primers. A rather challenging primer design involving selection of at least eight binding sites is thus simplified. Furthermore, Stem primers impose fewer limitations on the primer design in terms of stem length, orientation and distances between B1-B2 and F1-F2 sites. In contradiction to the postulated LAMP mechanism that relies on the involvement of displacement primers Stem primers can occasionally allow displacement primers not to be used at all, though it is not clear why this is so. This has a major implication for primer design, as it allows the ability to omit one displacement primer or even both, if necessary.

In conclusion, Stem primers offer a valuable additional tool for IVD assay development by easing restrictions on primer placement and design, whilst demonstrating required assay speed, sensitivity, and reproducibility at low copy number.

## Figures and Tables

**Table 1:** Sequences of oligonucleotides designed for *Clostridium difficile* targeting a toxin B.

Oligonucleotide	Sequence
CD-BIP	gggtcactcgtttcacttagctcgatggtgtaagtttaggtgcagc
CD-FIP	gcaatcattacttcatctttggggatagcggtatacctgctgaaattcctgc
CD-StemB	cctatcttagcttctatttcttgtc
CD-StemF	ggcagtaaatttaacaacagc
CD-DisplB	tacttcctacattatcgaagg
CD-DisplF	cgaagtacaagttcattgtttac
mCD-FIP	gcaggaatttcagcaggtataccaagctacaacctttgttgccttatctcg
mCD-StemB1	ctgccattatacctatcttagcttc
mCD-StemF1	tttaacaacagctacaactgc
mCD-StemB2	ccccaaagatgaagtaatgattg
mCD-StemF2	ctagtggatttagtatacttttagttcc
mCD-DisplF	tcagtttcaactaatgaaacatg

**Table 2:** Sequences of oligonucleotides designed for HIV-AT.

Oligonucleotide	Sequence
sHIV-BIP1	ctactacaggtggcaggttaaaatcactagtcacagtaattggagagcaatgg
sHIV-FIP1	gcatggacaagtagactgtagtccagccttctaaatgtgtacaatctagttgcc
HIV-StemB	cacagctggctactatttcttttg
HIV-StemF	gtcagctaaaaggagaagcc
HIV-DisplB	attagtcagtgctggaatcagg
HIV-DisplF	ggaataacttctgcttctatatatccactg
lHIV-BIP2	cacagctggctactatttcttttgctactcacagtaattggagagcaatgg
lHIV-FIP2	gtcagctaaaaggagaagccatgcccttctaaatgtgtacaatctagttgcc
HIV-LoopB	aggtggcaggttaaaatcac
HIV-LoopF	tggacaagtagactgtagtcc

**Table 3:** Sequences of oligonucleotides designed for *Listeria monocytogenes* internalin A.

Oligonucleotide	Sequence
LM-BIP	ccttcttttacaggcttagctggtttttcaaagaaacaaccaaagaagtgg
LM-FIP	ggaatttcagtacggataaaatgccgttttattatcaaacgttgctgtgtagc
LM-StemB	tcaaaccacccaacaaatg
LM-StemF	aaccggcggaactaaat
rLM-StemB	catttgttgggtggtttga
rLM-StemF	atttagttccgccggtt
LM-LoopB	catcgatttatatgcgcaat
LM-LoopF	cagtcaataaattcccagc
rLM-LoopB	gctgggaatttattgactg
rLM-LoopF	attgcgcatataaatcgatg
LM-DisplB	taatgctaagtttcatgtg
LM-DisplF	ataatctactgtttgagatg
